# Evaluation of the psychometric properties of the Brazilian version of the Oral Health Literacy Assessment in Spanish and development of a shortened form of the instrument

**DOI:** 10.1371/journal.pone.0207989

**Published:** 2018-11-29

**Authors:** Fernanda Maria Rovai Bado, Flávio Rebustini, Lisa Jamieson, Karine Laura Cortellazzi, Fábio Luiz Mialhe

**Affiliations:** 1 Department of Community Dentistry, University of Campinas, Piracicaba Dental School, Piracicaba, São Paulo, Brazil; 2 Department of Gerontology (EACH), University of São Paulo, São Paulo, Brazil; 3 Indigenous Oral Health Unit, The University of Adelaide, Adelaide, South Australia, Australia; University of Lleida, SPAIN

## Abstract

**Objective:**

The objective of this study was to investigate the psychometric properties of the Oral Health Literacy Assessment in Spanish (OHLA-S) for the Brazilian-Portuguese language using robust analysis and with the results disclose possibilities to develop a shorter and more valid instrument.

**Methods:**

OHLA-S is an oral health literacy instrument comprising a word recognition section and a comprehension section. It consists of 24 dental words. It was translated into the Brazilian-Portuguese language (OHLA-B) and its psychometric properties were evaluated in a random sample of 250 adults aged 20–59 years. To assess the dimensionality and factor structure were tested by means of Exploratory Factor Analysis (EFA) and Confirmatory Factor Analysis (CFA). Reliability was assessed using two indicators: Cronbach's alpha and McDonald’s Omega.

**Results:**

EFA and CFA demonstrated that the OHLA-B with 24 items did not present an adequate adjustment of the model, compromising its validity. In addition, reliability values at 0.50 for Cronbach's alpha and 0.67 for McDonald's omega were below the minimum acceptable rate of 0.70. As no support was found for the original structure, we decided to proceed with the withdrawal of individual items and successive reanalysis of the model until the indicators were adjusted in a shorter instrument. A new structure with 15 items produced an instrument with two dimensions and a better goodness of fit than the original instrument. The Alpha and Omega reliability index values increased to 0.83 and 0.80, respectively, and all scores were better in the OLHA-B with 15 items than in the instrument with 24 items.

**Conclusion:**

OLHA-B with the original structure composed by 24 items did not show acceptable construct validity. The shorter version with 15 items showed more promising results for assessing oral health literacy levels in the Brazilian population.

## Introduction

Health literacy represents the degree to which individuals have the capacity to obtain, process and understand basic information and the functioning of health services, skills that are crucial for individuals making appropriate health decisions [1]. Health literacy covers a range of skills that surpass the mere individual capacity to read and make notes, because it includes a set of skills that allows people to participate more actively in society and to increase their control of factors that can impact on their health [2,3].

Oral health literacy (OHL) is a relatively new field of study in dentistry. Research has demonstrated that low OHL is associated with limited knowledge and awareness of oral health, failure to keep appointments for dental care, lower levels of adherence of recommended oral health behaviors and poor oral health [4–6]. Therefore, OHL has been considered a new determinant of oral health and should be considered with more relevance in oral health research [7,8]. Most instruments developed to evaluate OHL have used word recognition and/or reading comprehension tests and focus on individuals’ ability to correctly pronounce oral health related vocabularies [9,10]. One example is the Rapid Estimate of Adult Literacy in Dentistry-30 (REALD-30) developed by Lee et al. [11]. The assumption of these word-recognition tests is that there is a strong correlation between reading and comprehension abilities, such as process text and understand its meaning, with clinically observed oral health. Another group of OHL tests measure a respondent’s ability to understand and apply written information, including numerical data, as demonstrated by the Test of Functional Health Literacy in Dentistry (TOFHLiD)[9,10].

The majority of instruments for measuring OHL have been developed in the English language and produced for application in the North American context [9,10,12]. In relation to the Portuguese language, while it is the world’s sixth most spoken language [13], very little is known about the OHL of people who speak this language. Two instruments originally developed in the English language were recently adapted and validated to measure functional OHL in the Brazilian adult population: the Brazilian Rapid Estimate of Adult Literacy in Dentistry (BREALD-30) [14] and the 20-item Rapid Estimate Adult Literacy in Medicine and Dentistry (REALMD-20) [15]. Despite their relevant contribution to a better understanding of OHL characteristics in the Brazilian context, it is known that the phonetic structure of the Portuguese and Spanish languages is highly regular; that is, one sound is usually represented by one letter and vice versa, which is not necessarily the case in the English language. This fact makes OHL instruments originally developed in the English language less appropriate for measuring functional health literacy in Spanish and Portuguese populations [16–18].

With the purpose of overcoming these problems, Lee et al. [16] developed the Oral Health Literacy Assessment in Spanish instrument (OHLA-S), which is characterized as being an adaptation of the same 30 dental words of REALD-30 [11] for the Spanish language with incorporation of a comprehension test. In the original OHLA-S validation study with 201 Spanish-speaking adults in the United States, authors tested three scoring systems for evaluating the psychometric properties of the instrument. After a series of validity asssessments to compare the results of the scoring systems, the authors recommended the use of the pronunciation and comprehension scoring method, which resulted in an instrument containing 24 words [16]. Despite these analyzes, the authors did not perform robust analyses of the psychometric characteristics of OLHA-S, such as assessment of its dimensionality [16].

Thus, due to the shortage of OHL measuring instruments for the Portuguese language, the development of further validation studies is important for the purpose of providing health professionals and researchers with fast and reliable tools for assessing these aspects in clinical encounters and in populations.

The objective of this study was to investigate the psychometric properties of the OHLA-S for the Brazilian-Portuguese language and use the results to develop a shorter and more valid instrument.

## Material and methods

Data for this study came from a cross-sectional investigation with adults who lived in the vicinity around a primary health care unit in Piracicaba, São Paulo, Brazil. This study was approved by the Research Ethics Committee of the Piracicaba Dental School under Protocol Number 140/2014 and conducted in accordance with the Declaration of Helsinki.

All participants signed a written Term of Informed Consent before commencement of data collection. Furthermore, before the study began we obtained permission from J.Y.Lee to translate and validate the instrument.

The initial translation and cross-cultural adaptation of OHLA-S into the Brazilian Portuguese language (OHLA-B) was done according to the protocol suggested by Guillemin et al.[19]: initial translation, back translation, review by a committee of experts and pretest. Initially, the OHLA-S was translated into the Brazilian Portuguese language by two independent translators, with an emphasis on conceptual translation rather than literal translation. Secondly, the Brazilian Portuguese version of OHLA-S underwent reverse translation into Spanish (back-translation) performed by two independent native Spanish language translators who had no access to the original instrument. Then, the two versions of the instrument were evaluated by a committee of experts, consisting of four dentists, including two researchers with translation experience. The review committee evaluated all stages of the process, the original and the final versions of the instrument, and by consensus, chose the best words for adapting them to the Brazilian cultural context [20,21]. Attached is an appendix (S1 Table) showing the 30 items of REALD-30 and the 24 items appearing on the recommended OHLA-Spanish (OHLA-S) and on the OHLA-Brazilian (OLHA-B) version.

In the pre-testing stage, the OHLA-B was administered to a convenience sample of 20 adults with a mean age of 42.5 years (SD: 14.1) and a mean of 11.4 years of schooling (SD: 4.3). The participants were instructed to say "I do not understand" if they not understand any words of the instrument. The results of the cultural adaptation demonstrated that the Brazilian version of OHLA-S was well understood by 20 individuals in this study who participated in the pre-test. The level of misunderstanding did not exceed 3 participants in any of the 24 questions [20,21].

After the appropriate translation and cross-cultural adaptation of OHLA-B, the instrument was applied by a single investigator (FMRB), who had previously undergone a training process for the interview protocol. A sample size of 250 participants was calculated based on minimum acceptable ratio of 10 respondents for each item of the instrument in psychometric studies [22]. The interviews were held in the homes of the participants aged between 20 and 59 years, who lived in the vicinity of a primary health care unit in Piracicaba, São Paulo, Brazil. Data available from the Dryad Digital Repository: https://doi.org/10.5061/dryad.jc6dt

A simple random sampling process using a lottery draw method was used to select the participants who fulfilled all the inclusion criteria based on data from the primary care information, a government repository tool for health teams planning and monitoring service delivery in primary care. To this end, 282 individuals were contacted and 250 accepted to participate.

After their acceptance, the researchers went to the volunteers’ homes to collect data. To be considered eligible for the study, individuals had to present self-reported ability to read and speak the Brazilian Portuguese language; have no diagnosis of dementia, visual or hearing impairment; and to have not used alcohol or drugs at the time of the interview. Excluded from the sample were individuals who were illiterate.

The instrument presented a pronunciation test and a comprehension test. For the pronunciation test, the participant was shown a card containing a dental vocabulary word that he/she had to read out aloud. At the time, the researcher checked if the pronunciation was correct. Then the researcher applied the word comprehension test, in which the participant had to choose between two other words, one that was meaningfully associated with the OHLA-B word initially pronounced. The individual had the option to say “I don´t know”. The total time of OHLA-B application was 3 to 5 minutes.

In the original OLHA-S development and validation article, Lee et al [11] tested three scoring methods for evaluating the OHLA-S results and recommended the use of the named “Pronunciation and Comprehension” (P and C) one because it achieved the best balance between validity and reliability. In this method, a score of 1 is assigned when the results of the pronunciation and association tests were both correct; and a score of 0 when either any of the two was incorrect. We used this criteria recommended by the authors for data analysis.

### Data analysis

For the analysis of the psychometric properties of OLHA-B we used the Exploratory Factor Analysis (EFA) and the Confirmatory Factor Analysis (CFA). Factorial analysis requires the fulfillment of several steps, such as inspection of data, method of factor analysis, factor retention technique, factor rotation analysis and factor loading cut-off level [23,24].

Exploratory Factor Analysis (EFA) and Confirmatory Factor Analysis (CFA) assessments were performed through dimensionality and structure testing which was performed with Robust Parallel Analysis (RPA) by means of Optimal implementation of Parallel Analysis (PA) with minimum rank factor analysis which minimizes the common variance of residues [25]. The robustness of the test was determined from the association of a bootstrap with a sample extrapolation to 5000. The estimation of the tetrachoric matrix was performed using the Bayes Modal Estimation [26]. The dimensionality in the EFA (unrestricted model) was tested by Parallel Analysis which has been considered one of the most robust and accurate techniques for dimensionality testing [24,27–29]. The extraction of the factors in both EFA and CFA was done by the RULS (Robust Unweighted Least Squares) technique that reduces the residues of matrices [30] and is more robust in non-normal data [31].

If the instrument demonstrated more than 1 dimension, the non-orthogonal technique [32] was adopted, which is more appropriate for psychosocial latent variables [33]. We used Promax as rotation technique.

It was adopted if the following criteria were met [34]: UNICO (Unidimensional Congruence > 0.95), ECV (Explained Common Variance > 0.80) and MIREAL (Mean of Item Residual Absolute Loadings < 0.30).

Regarding the quality parameters of the instruments, the explanatory variance of the instrument should be around 60% [33]. The cut-off of fator loads of 0.30 are recommended when the sample has at least 300 individuals [33], but the model should look for factor loads above 0.50 [33, 35]. As the sample was 250 participants the cut-off of factorial loads was 0.35 [33]. The communalities (h^2^) should have values above 0.40 [36]. The maintenance or withdrawal of an item from the model depends on the magnitude of the communalities, factor loads, sample size, the degree that the item can measure the factor [37] and the lack of cross-loading [33].

In relation to the CFA adjustment rates, the indices recommended by Hair [33] were adopted as follows: NNFI (Non-Normed Fit Index ≥ 0.95); CFI (Comparative Fit Index ≥ 0.95), GFI (Goodness Fit Index ≥ 0.95), AGFI (Adjusted Goodness Fit Index ≥ 0.95), RMSEA (Root Mean Square Error of Approximation ≤ 0.08) and RMSR (Root Mean Square of Residuals ≤ 0,8).

Reliability of data was evaluated by two indicators: Cronbach’s Alpha [38] and McDonald’s Omega [39]. The adoption of two indicators seeks to increase the reliability of the interpretation, since reliability inconsistencies have occurred through Cronbach's Alpha [40–42].

Replicability of the construct and the quality of the factorial solution was evaluated by the Generalized G-H Index [43] that evaluates how well the factor is represented by the items. The G-H index measures the maximum proportion of the factor variance that can be measured by the items and two properties of the factorial analysis: a) the quality of the items as indicators of the factor and; b) the expected replicability of the solution between the studies. Hancock and Mueller [43] proposed a cut of 0.70 and, more recently, Rodriguez et al [44] proposed a minimum of 0.80.

The analyses were performed with SPSS 23, AMOS 23 and Factor 10.8.

## Results

The sample suitability indices presented good conditions for the factorial analysis: matrix determinant value of 0.019, Kaiser-Meyer-Olkin (KMO) of 0.79 and Bartlett's sphericity of 941.7 (degrees of freedom = 253) and p <0.001.

The first analysis had problems because item 1 (sugar) did not present variance due to the fact that all respondents had both pronunciation and the comprehension correct for this item. The analysis was redone without item 1 and the Parallel Analysis indicated the presence of two dimensions with an explained variance of 29.1%. The multidimensionality was confirmed by the values of UNICO = 0.60, ECV = 0.55 and MIREAL = 0.26, all them reaffirming that the items of the instrument are reported in a multidimensional form. The eigenvalue criterion above 1 (Kaiser's criterion) reported 8 dimensions with eigenvalues between 3.16 and 1.02. An explained variance of 29.1% indicated an initial model of low explanatory power and probably with low adjustment. Eleven of the 24 items had factorial loads below 0.35 on both factors. For the 13 items the factorial loads ranged from 0.35 to 0.67.

The communalities ranged from 0.01 to 0.45 and only 3 items had values above 0.30. This result explains the low explained variance of the model.

Reliability analysis demonstrated values of 0.50 for Cronbach's alpha and 0.67 for McDonald's omega which are below the minimum acceptable rate of 0.70. The non-fit of the model is reinforced by its instability related to the G-H index, which was 0.77 and 0.75, respectively for the first and second dimension. The G-H index pointed to the impossibility of the initial model to remain stable in different groups of populations.

In addition to the EFA indicators, CFA's model adjustment quality scores also presented problems all below the floor of 0.95 (0.85 to 0.91). Only the RMSEA and RMSR were below the required of 0.08. Therefore, both the EFA and the CFA indicators were not considered adequate and satisfactory.

From these results, we decided to withdraw individual items and to reanalyse the model until the indicators were adjusted in order to develop a shorter version of OHLA-B. It was necessary to remove 9 items (1, 2, 3, 4, 7, 8, 13, 15 and 24) in order to improve the model fit. This procedure resulted in factorial loads ranging from 0.51 to 0.92, which met the criterion of good quality (> 0.50) and the communalities improved their values varying from 0.27 to 0.86 (Table 1).

**Table 1:** Factorial loads and communalities (h^2^) comparison between the initial model (OHLA-B with 24 items) and the adjusted model (OHLA-B with 15 items).

	Initial Model			Adjusted Model		
Item	Factor 1	Factor 2	h^2^	Factor 1	Factor 2	h^2^
1 Sugar	-	-	-			
2 Smoking	0.01	0.13	0.01			
3 Brush (verb)	-0.20	0.67	0.45			
4 Pulp	0.14	-0.01	0.01			
5 Braces	0.26	-0.05	0.03	0.01	0,51	0,27
6 Genetics	0.11	0.56	0.24	0,66	0.19	0,51
7 Restoration	-0.05	0.21	0.04			
8 Bruxism	0.33	0.39	0.18			
9 Abscess	0.39	-0.01	0.10	0.11	0,62	0,42
10 Extraction	-0.03	0.58	0.38	0,92	-0.20	0,86
11 Denture	-0.01	0.36	0.07	0,56	0.03	0,32
12 Enamel	0.07	0.26	0.03	0,58	0.16	0,39
13 Dentition	-0.02	0.18	0.01			
14 Calculus	0.51	-0.00	0.22	0.08	0,71	0,53
15 Gingiva	-0.14	0.40	0.16			
16 Maloccusion	0.33	-0.01	0.06	-0.03	0,63	0,40
17 Incipient	0.33	-0.01	0.07	0.11	0,59	0,37
18 Caries	-0.08	0.39	0.12	0,58	-0,20	0,36
19 Periodontal	0.45	0.08	0.15	0.15	0,66	0,48
20 Hypoplasia	0.75	-0.11	0.45	-0,23	0,90	0,82
21 Halitosis	0.09	0.54	0.20	0,73	0.13	0,58
22 Analgesia	0.01	0.25	0.02	0,56	0.07	0,33
23 Fistula	0.48	-0.01	0.19	-0.01	0,68	0,47
24 Temporomandibular	0.26	0.21	0.06			

The improvement of these indicators raised the explained variance of the model to 57.5%. The reliability of model demonstrated improvements: Cronbach's alpha increased to 0.83 and McDonald's Omega to 0.80. In addition, the reproductive index (G-H index) increased to acceptable levels above 0.80 with two dimensions at 0.91. The correlation between the factors was r = 0.17 in the final solution, while in the initial extraction it was r = -0.01

The set of improvement in the indexes found in the EFA also occurred in the CFA. All quality indices of the model were above the established quality criteria, ranging from 0.95 to 0.87, the RMSEA was 0.06 and the RMSR was 0.05. Table 2 shows the EFA and CFA indicators for the initial model and the adjusted model. The analyzes demonstrated the unfeasibility of the original unidimensional model of the instrument presented by Lee et al. [16], indeed, there was no testing of dimensionality in their article.

**Table 2:** Summary of the initial and adjusted model characteristics.

	Index	Technique	Initial model	Adjusted model
Exploratory	Adequacy of correlation matrix^a^	Determinant of the matrix	0.01	0.09
		Bartlett	941.7(df = 253)*	563.9 (df = 105)*
		KMO (Kaiser-Meyer-Olkin)	0.79	0.78 (0.78–0.82)
	Dimensions (Parallel analysis)		2	2
	Explained Variance by eigenvalues		39.10%	54.08%
	Explained Variance		29.10%	57.70%
Dimensionality	Unidimensional Congruence (UNICO)		0.60	0.80
	Explained Common Variance (ECV)		0.55	0.61
	Mean of item residual absolute loading (MIREAL)		0.26	0.42
Confirmatory	Robust Mean and Variance-Adjusted Chi Square (X^2^/df)		1.69	1.98
	Non-Normed Fit Index (NNFI)		0.85	0.96
	Comparative Fit Index (CFI)		0.87	0.97
	Goodness of Fit Index (GFI)		0.91	0.96
	Adjusted Goodness of Fit Index (AGFI)		0.89	0.95
	Root Mean Square Error of Approximation (RMSEA)		0.05	0.06
	Root Mean Square of Residuals (RMSR)		0.07	0.07
Reliability	Standardized Cronbach's Alpha		0.57	0.83
	McDonald's Omega		0.67	0.80
	Construct reliability–GH Latent index		0.77 and 0.75^a^	0.91 and 0.91^a^

* p < 0.001

^a^—G-H for dimension 1 and 2, respectively

## Discussion

This study demonstrated that the psychometric properties of the Brazilian-Portuguese version of the OLHA-S with 24 items using the recommended pronunciation and comprehension scoring method by the authors of the original instrument did not shows acceptable validity and reliability. These results were not observed in original validation study of OHLA-S because in the original validation study the authors did not test the dimensionality of the instrument by factor extraction, factor loading, communalities or other information to confirm the goodness of fit, assuming that it was unidimensional [16].

In view of the unsatisfactory psychometric findings with OLHA-B with 24 items, we reanalyzed the model to obtain a shorter version of the instrument with more promising results. We found that an instrument with 15 items showed more acceptable psychometric characteristics for use on the Brazilian population.

Furthermore, our findings demonstrated that OHLA-B with 15 items presented high reliability, as Cronbach´s Alpha coefficient (α = 0.83) was better than found by Lee et al. [16] (α = 0.70) when they developed the original OHLA-S with 24 items, and Villanueva et al. [45] (α = 0.784) in a recent assessment of the Spanish Oral Health Literacy Scale (SOHLS). On the other hand, the Cronbach´s Alpha value was lower than the Brazilian version of REALD-30 (α = 0.88–0.89) [14]. However, the comparison between Alpha values could be imprecise because there is important scientific literature showing that the Alpha is highly influenced by the number of items in the instrument, and thus its precision can be artificially increased [39–43]. In addition, according to Sijtsma [46] Alpha “only is a lower bound to the reliability and not even a realistic one” and does not provide information on the internal structure of the test. In order to overcome these limitations, the reliability of OLHA-B 15 was also assessed by McDonald's omega [39] to increase the accuracy of the data. We found that the Omega values were better (0.80) in OLHA-B with 15 items than that with 24 items (0.67).

Prior to conducting factor analyses, the item “sugar” was withdrawn because it showed no variability (all 250 subjects had the same score). This fact was also observed in a two-stage study of REALD-30 [47] and in a study that evaluated the validity REALD-30 in Saudi Arabia [48], demonstrating that this word did not contribute to the psychometric properties of these tools.

The necessity of changes in the original instrument for measuring OHL in order to improve their psychometric properties was presented in previous articles [14,49,50]. In the draft of OLHA-S in the original validation study, the authors initially tested the instrument with the same 30 dental words as those in the REALD-30 [16]. However, after they had tested three different scoring methods, it was recommended to use one that with 24 items [16]. We followed the same research methodology as the original article, but with more robust statistical analysis.

Parallel analysis showed the presence of two factors in OLHA-B with 15 items. The seven dental terms that loaded in the first factor (genetics, extraction, denture, enamel, caries, halitosis and analgesia) were interpreted as commonly used dental terms by patients in day life and also obtained from toothpaste advertisements broadly disseminated in Brazilian media. On the other hand, the eight dental terms loaded in the second factor (braces, abscess, calculus, malloclusion, incipient, periodontal, hypoplasia and fistula) are more difficult to understand by the lay public and are generally used by oral health professionals for specific dental conditions. Therefore, the division between the factors is coherent in relation to its content and this follows the same pattern of REALD-30 studies in which the instrument presents two factors, with the second domain comprising the most difficult words [11,48,49]. In addition, the present instrument demonstrated the two essential characteristics of REALD-30 and OLHA-S: words related to adverse oral health conditions and arranged in ascending order of reading difficulty [11,16].

In the OLHA-B with 15 items, two factors explained 57.5% of the total variance. This result is better than other word recognition-based tools for measuring oral health literacy translated and validated for the Brazilian-portuguese language. In the Brazilian version of REALD-30 (BREALD-30) a minimum of seven factors were necessary to explain 50% of total variance [14] and in the Brazilian version REALMD-20, the first four factors accounted for 52.1% of the variance [15]. This suggests that additional constructs could exist in these instruments, which should be further investigated.

It is important to highlight that the authors who created the REALD-30, the instrument that was used as the basis for developing OLHA-S, proposed its revision four years after, transforming it into a more efficient and easier-to-use two-stage scale [47]. The authors found that the new scale, one third of the length of the original REALD-30 (only 10 items), showed a correlation of 0.96 with the original instrument, demonstrating the appropriateness of the new scoring system. Therefore, the development of future studies with OLHA-B in another population is essential for testing of its psychometric properties tocorroborate, or not, our findings.

A strength of this study was its consistent and robust protocols to test the dimensionality, factor structure and reliability of the instrument, by combining EFA and CFA showing how changes of items could cause important changes in the results for adjustment of the model. To the best of our knowledge, there are few articles that have evaluated the psychometric characteristics of a functional oral health literacy instrument by means of these analyses. These limitations were recently exposed in two review articles on the use of instruments for measuring oral health literacy [9,10].

However, despite the advances this study contributes to the oral health literacy field, it has some limitations. The use of a sample size that lived in the vicinity around a primary health care unit, although comprised of individuals from various socioeconomic levels, limited the generalizability of the results to the whole city, so that further studies are required with a representative sample of the population to determine the external validity of the results. In addition, our study involves a population somewhat different than the populations studied by Lee and her colleagues, a fact that compromises the direct comparability of the data. Furthermore, the assessment of validity should be interpreted cautiously since the evaluation was confined to one approach and responsiveness to change of the instrument could not be tested due to this being a cross-sectional study. Nevertheless, further studies among larger and more representative sample of populations are required because the sample of our study did not allow us the accomplishment of cross-validation tests impeding the partition of the sample so that we could carry out a training set and a test set. This would allow a better support for the indexes of reliability and model fit, as well as the reduction of any bias in sub-populations. Even so, the results were supported by contemporary statistical techniques that pointed out that the 15-item model works better than the 24-item model in the Brazilian population.

Finally, it is important to consider that OHLA-B is an instrument for the measurement of functional oral health literacy and only evaluates the reading, pronunciation and comprehension literacy skills, but not the numeracy and critical literacy skills, important domains in the field of health literacy.

In conclusion, this study showed that OLHA-B with the original structure composed by 24 dental items did not show acceptable construct validity. The shorter version with 15 items showed evidence of construct validity by both EFA and CFA and more promising results for measurnig oral health literacy in the Brazilian population.

## Supporting information

S1 Table Comparison between REALD-30, the recommended OHLA-S version with 24 items and the initial version of OHLA-B. (DOCX)
