# Supplementary material for: Evaluation of the psychometric properties of the Brazilian version of the Oral Health Literacy Assessment in Spanish and development of a shortened form of the instrument
Source: PLoS One. 2018 Nov 29;13(11):e0207989. doi: 10.1371/journal.pone.0207989 (PMC6264477; doi:10.1371/journal.pone.0207989)
Supplement: S1 Table — (DOCX) [file pone.0207989.s001.docx]

**Supporting information**

**S1 Table: Comparison between REALD-30, the recommended OHLA-S version with 24 items and the initial version of OHLA-B.**

|  |  |  | **REALD-30** |  |  |  |  |  | **OHLA-S** | **OHLA-B** |  |
| --- | --- | --- | --- | --- | --- | --- | --- | --- | --- | --- | --- |
| 1 |  |  | Sugar |  |  |  |  |  | Azúcar | Açúcar |  |
| 2 |  |  | Smoking |  |  |  |  |  | Fumar | Fumar |  |
| 3 |  |  | Floss |  |  |  |  |  |  |  |  |
| 4 |  |  | Brush |  |  |  |  |  | Cepillar | Escovar |  |
| 5 |  |  | Pulp |  |  |  |  |  | Pulpa | Polpa |  |
| 6 |  |  | Fluoride |  |  |  |  |  |  |  |  |
| 7 |  |  | Braces |  |  |  |  |  | Frenos | Braquetes |  |
| 8 |  |  | Genetics |  |  |  |  |  | Genética | Genética |  |
| 9 |  |  | Restoration |  |  |  |  |  | Restauración | Restauração |  |
| 10 |  |  | Bruxism |  |  |  |  |  | Bruxismo | Bruxismo |  |
| 11 |  |  | Abscess |  |  |  |  |  | Absceso | Abscesso |  |
| 12 |  |  | Extraction |  |  |  |  |  | Extracción | Extração |  |
| 13 |  |  | Denture |  |  |  |  |  | Dentadurapostiza | Dentadura |  |
| 14 |  |  | Enamel |  |  |  |  |  | Esmalte | Esmalte |  |
| 15 |  |  | Dentition |  |  |  |  |  | Dentición | Dentição |  |
| 16 |  |  | Calculus |  |  |  |  |  | Cálculo | Cálculo |  |
| 17 |  |  | Gingiva |  |  |  |  |  | Encía | Gengiva |  |
| 18 |  |  | Malocclusion |  |  |  |  |  | Maloclusión | Má-oclusão |  |
| 19 |  |  | Incipient |  |  |  |  |  | Incipiente | Incipiente |  |
| 20 |  |  | Caries |  |  |  |  |  | Caries | Cárie |  |
| 21 |  |  | Periodontal |  |  |  |  |  | Periodontal | Periodontal |  |
| 22 |  |  | Sealant |  |  |  |  |  |  |  |  |
| 23 |  |  | Hypoplasia |  |  |  |  |  | Hipoplasia | Hipoplasia |  |
| 24 |  |  | Halitosis |  |  |  |  |  | Halitosis | Halitose |  |
| 25 |  |  | Analgesia |  |  |  |  |  | Analgésico | Analgésico |  |
| 26 |  |  | Cellulitis |  |  |  |  |  |  |  |  |
| 27 |  |  | Fistula |  |  |  |  |  | Fístula | Fístula |  |
| 28 |  |  | Temporomandibular |  |  |  |  |  | Temporomandibular | Temporomandibular |  |
| 29 |  |  | Hyperemia |  |  |  |  |  |  |  |  |
| 30 |  |  | Apicoectomy |  |  |  |  |  |  |  |  |
